# Mediterranean diet: a potential player in the link between oral microbiome and oral diseases

**DOI:** 10.1080/20002297.2024.2329474

**Published:** 2024-03-19

**Authors:** Giuseppina Augimeri, Giovanna Caparello, Ippolito Caputo, Rodolfo Reda, Luca Testarelli, Daniela Bonofiglio

**Affiliations:** a Department of Pharmacy, Health and Nutritional Sciences, University of Calabria, Rende, Italy; b Department of Oral and Maxillo Facial Sciences, Sapienza University of Rome, Rome, Italy; c Centro Sanitario, University of Calabria, Arcavacata di Rende, Italy

**Keywords:** Mediterranean Diet, oral microbiome, oral diseases, periodontal diseases, chronic metabolic diseases, dental plaque, biofilm, nutrients, oral health implications

## Abstract

**Background:**

The oral microbiome is a complex and dynamic assemblage of microorganisms that colonize different sites of the oral cavity maintaining both oral and systemic health. Therefore, when its composition is altered, oral diseases occur. Among oral inflammatory pathologies, periodontal diseases affect the tissues surrounding the teeth, representing the main cause of tooth loss and one of the most important threats to the oral health. Lifestyle and eating habits influence the composition of the human oral microbiota and the development and progression of oral diseases. In this context, the Mediterranean Diet (MD) model, comprising both healthy dietary choices and lifestyle, is linked to the prevention of several metabolic and chronic-degenerative pathological processes, including oral diseases. Indeed, the MD is a plant-based diet, enriched of anti-inflammatory and antioxidant nutrients, which may induce beneficial effects against dental caries and periodontal diseases.

**Aim:**

This review summarizes the role of the oral microbiome in the development of the oral diseases and the potential of MD in modulating the oral microbiome leading to implications for oral health.

**Conclusions:**

The data collected highlight the need to promote the MD pattern along with the correct hygiene habits to prevent the development of oral diseases.

## 3 key messages

- The oral microbiome is a complex and dynamic assemblage of microorganisms that colonize different sites of the oral cavity maintaining both oral and systemic health.
- The Mediterranean Diet (MD) model, comprising both healthy dietary choices and lifestyle, is linked to the prevention of several metabolic and chronic degenerative pathological processes, including oral diseases.
- The MD may represent a potential player in the link between oral microbiome and oral diseases.

## Introduction

Oral microbiome, representing the second largest and diverse microbiome after the gut, refers to the collective microorganisms seeded in the oral cavity. It accounts for approximately 700 species of bacteria, fungi, viruses, archaea, and protozoa [1], which colonize teeth, tongue, cheeks, gingival sulcus, tonsils, hard and soft palate, as well as the surfaces of the oral cavity [2,3]. The tropism of each species of microorganism to the surface of the mouth, i.e. the ability of the microorganism to interact positively with the host surface, depends on the expression of adhesion proteins, which recognize the receptors on the oral surface and, depending on the substrate, it expresses a different pathogenic potential [4]. The Human Microbiome Project has identified an oral core microbiome, shared among individuals that changes across a life-history stages of the host [5]. In addition, a variable microbiome unique for each subject, which depends on the lifestyle and genotypic determinants, has been found [6]. The oral microbiota interacts with the host to reflect the immunity and metabolic status through two-way communication along the oral cavity and the systemic organs. These phenomena are extremely important in maintaining the balance of the microbiota, such as the ratio of ‘commensals’ *Veilonelle*, which produce vitamin K used by *B. melaninogenicus*, or the ‘antagonistic’ relationship of different *Streptococci,* which produce bacteriocins capable of being active on bacteria of the same or different species [7]. Thus, the oral cavity is one of the most important interaction windows between the human body and the environment [8]. Alterations of the oral microbiome led to a wide range of pathological conditions, including oral and systemic diseases [9,10]. Several exogenous factors, such as diet, can influence the composition of the microbiome [11], suggesting the important role of a healthy dietary pattern in maintaining eubiosis of the oral microbiota.

The Mediterranean Diet (MD) is a plant-based diet, which has been recognized as one of the healthiest dietary patterns worldwide [12]. MD showed beneficial effects in the prevention and development of several diseases, including the non-communicable diseases (NCDs), such as diabetes, metabolic syndrome, cardiovascular and neurodegenerative diseases, and cancer [13]. It has been found that oral diseases are associated with the most common NCDs [14]. Thus, the oral health needs to be promoted in order to decrease the development of NCDs. Interestingly, the MD showed positive effects against oral diseases because of the bioactive molecules characterizing the Mediterranean foods [15,16]. However, it is still not completely understood whether the MD might modify the oral microbiome composition affecting the oral diseases.

Here, we summarize the role of the oral microbiome in the development of the oral diseases and the potential of MD in modulating the oral microbiome leading to implications for oral health.

## The MD pattern

The MD is one of the most studied dietary patterns associated with many benefits for human health worldwide [12]. The origins of the traditional MD were found in the civilizations surrounding the Mediterranean Sea, closely reflecting the social behaviors and lifestyles of that geographic area [17]. Although the different food cultures vary according to the Mediterranean region, a basic Mediterranean dietary pattern can be identified, making the MD a diversified heritage [17]. The MD eating pattern is characterized by abundance of plant food consumption represented by fruits, vegetables, breads and other cereals (traditionally minimally refined), legumes, nuts, seeds, and extra virgin olive oil (EVO) [18]. MD pattern also includes the consumption of moderate amounts of eggs and dairy products (principally low-fat cheese and yogurt), low-to-moderate amounts of fish and poultry, low amounts of red meat, and modest consumption of wine, normally with meals [19,20]. The MD food recommendations are graphed using the typical MD pyramid, which indicates the weekly and daily consumption frequencies of each food of the MD pattern [20]. Particularly, the food groups that should be consumed in greatest quantities appear in the largest section of the pyramid; conversely, foods that should be consumed in small quantities are represented in the narrowest part of the pyramid [18]. According to the European and US Dietary Guidelines, a healthy diet is based on approximately 55–60% of carbohydrates mainly represented by high-complex carbohydrate intake, like grains and legumes, 10–15% of proteins with lower consumption of animal than plant origin proteins, and 25–30% of total dietary fat intake. Regarding fats, no more than 7–8% of total calories should be saturated fats and cholesterol less than 300 mg/day with moderate consumption of EVO as main source of monounsaturated fatty acids. In addition, both omega-3 and omega-6 polyunsaturated fatty acids have to be ingested through diet, predominantly from marine organisms or from seeds [21].

**Figure 1.**
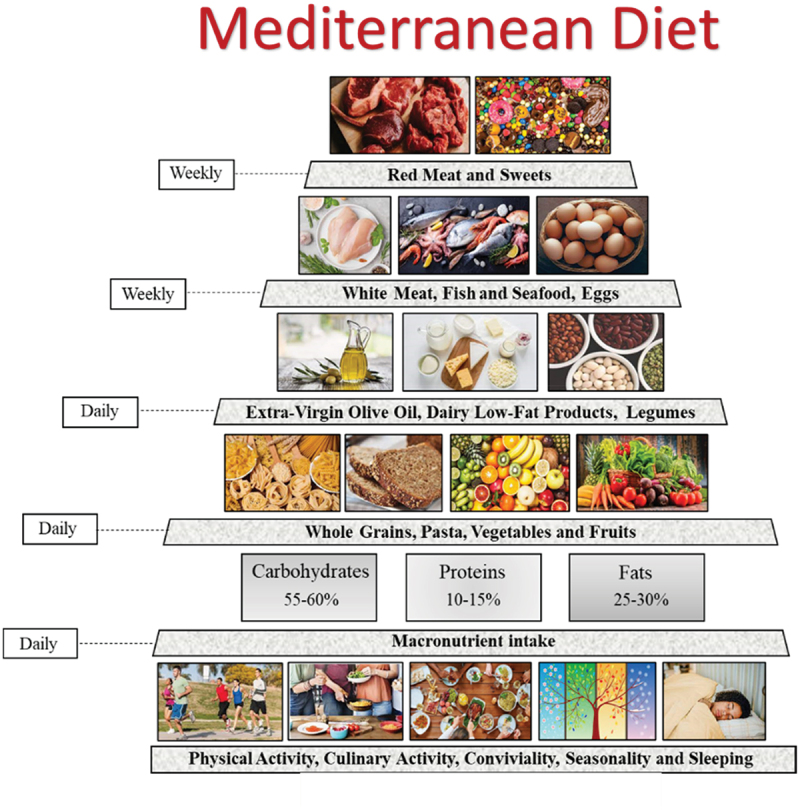
Mediterranean Diet pyramid and daily recommended macronutrient intake.

After the recognition of the MD as an Intangible Cultural Heritage of Humanity by UNESCO in 2010, new food recommendations have been proposed resulting in a new revised graphic representation of the pyramid [22]. Particularly, the scientific experts of the Mediterranean Diet Foundation’s International Scientific Committee together with the Forum on Mediterranean Food Cultures, giving the changes in the lifestyle, dietary, socio-cultural, and environmental habits of the global population, have adapted the food recommendations considering the various geographical, socio-economic, and cultural contexts of each Mediterranean region [18]. As a consequence, cultural and lifestyle elements were introduced in the MD pyramid as important elements of the MD pattern. These concepts include physical activity, adequate rest, seasonality, biodiversity, traditional and local food products, and socialization (Figure 1).

Interestingly, the MD has been proposed as a ‘gold standard’ diet not only due to its major health and nutrition benefits but also its lower environmental impact and richness in biodiversity, sociocultural food value, and its positive local economic returns. Indeed, since MD is mainly a plant-based diet with low consumption of animal products, it is characterized by a smaller water footprint and lower greenhouse gas emissions compared with other current dietary patterns [23–27].

Despite the recommendation of the MD pattern, about 70% of the population in Mediterranean countries exceeds in the consumption of saturated fats, sodium, and added sugars, highlighting the need to promote the adoption of a healthy eating pattern in the general population [28].

## The beneficial effects of the tablet against NCDs

NCDs, including diabetes, cardiovascular and neurodegenerative diseases, as well as cancers, are global health problems since they have an important impact on the quality and life expectancy and cause death and disability to millions of people worldwide [22]. NCDs share four major risk factors: smoking, physical inactivity, alcohol consumption, and unhealthy diets [29]. Controlling these risk factors represents the goal to reduce the burden of NCDs [29,30].

Since the pioneer Seven Countries Study by Ancel Keys [31], many scientific evidences highlighted the protective effects of the MD on metabolic as well as chronic and degenerative diseases [32–37]. Indeed, it has been demonstrated that several healthy foods and nutrients included in MD have been inversely associated with incidence of type-2 diabetes [38–40]. Similarly, a high adherence to the MD pattern is associated with a significant reduction in mortality from cardiovascular and neurodegenerative diseases and cancer [41–45]. Particularly, a critical review by Martinez Gonzales et al. reported strong evidences supporting MD as an ideal approach for cardiovascular health [46]. Findings from a recent randomized clinical trial, the CORDIOPREV study, demonstrated that MD prevented major cardiovascular events respect to a low-fat diet, suggesting the adoption of this eating model in long-term secondary prevention of cardiovascular disease [47]. Interestingly, a high adherence to the MD showed beneficial effects in population regardless ethnic groups, geographical, and socio-cultural settings [48]. For example, a lower score for MD adherence was found in inhabitants from the southeastern Spain compared to other Spanish areas, which was strongly associated with increased prevalence of hypertension [49]. Interestingly, a study conducted in a cohort of Anglo-Celts and Greek-Australians living in Melbourne showed that MD adherence was associated with longer survival even in a non-Mediterranean country [50]. Data on the degree of MD adherence across adults from different United States regions demonstrated that the adherence to MD was geospatially different affecting the prevalence of NCDs [51].

The beneficial effects of the MD foods depend on the presence of phytochemical compounds, which can modulate different biological functions in normal and altered cells. Several molecules found in the MD foods exert antioxidant, anti-inflammatory, antibacterial, pro or antiapoptotic, and vasodilatory activities, mediating anti-tumor, cardioprotective, and neuroprotective actions [52]. For example, it has been demonstrated that polyphenols and other phenylpropanoids present in fruits, vegetables, cereals, legumes, olive oil, cocoa, and plants-based drinks (for example tea, wine, and coffee), acting independently or synergistically, prevent the onset of several NCDs [53,54].

A meta-analysis of 2650 patients showed that the MD provided a more robust reduction in cardiovascular disease risk factors and inflammatory markers because of the consumption of the marine omega-3 fatty acids, which intake is widely recommended in the MD pattern [55]. The mechanisms by which fish prevent cardiovascular diseases are, at least in part, understood and include the inhibition of inflammation, oxidation, and coagulation processes, which lead to the improvement of the lipid profiles and the reduction of the blood pressure [56–58]. Particularly, the biomolecules responsible for these activities are mainly represented by omega-3 fatty acids, which modulate the activation of intracellular signaling pathways involved in different pathophysiological conditions [59].

Alzheimer’s, Parkinson’s, and other forms of cognitive decline extending from mild-cognitive impairment to vascular dementia represent some of the most concerning neurodegenerative diseases involving over million people [60,61]. Several Mediterranean dietary components have been studied to evaluate whether their potential antioxidant, anti-inflammatory, and vasodilatory effects may modify cognitive decline, as revised by Dominguez et al. [62]. Among the micronutrients of the MD foods, vitamins B6, B12, C, D, E, beta-carotene, folic acid, omega-3 fatty acids, zinc, and magnesium have shown neuroprotective effects and may be considered as optimal compounds for the maintenance of brain health [63].

A meta-analysis including over 1.7 million participants have reported that high adherence to MD was associated with a significantly lower risk of all-cause of mortality for different types of cancer, including breast, gastrointestinal (such as liver and pancreas), head and neck, prostate, and pulmonary cancer [64]. Particularly, data from PREDIMED trial have demonstrated that the highest nuts consumption, typical component of MD model, was associated with a 40% decrease in cancer mortality compared to the lowest consumption, as well as a long-term dietary intervention with an MD supplemented with EVO may reduce the risk of breast cancer [65,66].

### MD and oral diseases

Among NCDs, chronic oral diseases are largely preventable pathological conditions, which represent a serious health and economic burdens worldwide [67]. In 2017, approximately 50% of the world’s population suffered from oral diseases [68]. Specifically, dental caries, periodontal diseases, teeth loss, and cancers of the lips and oral cavity are the most prevalent and concerning issues [68]. Adhering to a healthy eating pattern might prevent the onset of this type of disorders [69]. In particular, the MD rich in anti-inflammatory and antioxidant foods has beneficial effects in patients with periodontal inflammation. Indeed, Bartha et al. have demonstrated a significant decrease in the bleeding on probing, gingival index, and periodontal inflamed surface in periodontal inflammatory patients following the MD for 6 months [70]. In contrast, an increased gingival inflammatory response was observed in people following a Western-type diet, characterized by high consumption of refined grains, sugar, and omega-6 fatty acids [71,72]. Interestingly, reduced serum omega-6 levels were detected, determining a lower omega-6/omega-3 ratio, which may be responsible for the improvement in their gingival inflammatory parameters [73]. Supporting the importance of consuming omega-3 fatty acids, results from a cross-sectional study demonstrated that consumption of olive oil showed protective effects against periodontitis in young Moroccan, although no significant association was found between adherence to the MD and periodontitis [74]. As above reported, the MD is not only a diet, but it is a healthy lifestyle based on a balanced eating habit and regular physical activity [75]. Marruganti et al. demonstrated an inverse correlation among the MD adherence, physical activity, and periodontitis onset. In particular, subjects with lower MD adherence and moderate physical activity showed worse biometric and inflammatory parameters along with increased mobility and teeth loss due to periodontitis than higher MD adherers [76]. The negative correlation between Stage-III/IV periodontitis and the MD adherence in patients was mainly due to their reduced consumption of wholegrain products. Of note, lower adherers to the MD declaring lower wholegrain product intakes showed eightfold increase of Stage-III/IV periodontitis, along with decreasing insulin sensitivity and increasing low-grade systemic inflammation [77]. It is therefore possible to suppose, from this point of view, a correlation between periodontal pathology, the anti-inflammatory power of various foods, antioxidant molecules contained in them, which can have an action not only through topical application but also systemically through the blood circulation. Indeed, in a recent systematic review, the positive effects of the natural phytochemicals, due to their documented antioxidant properties, have been correlated with improvements in periodontitis [78]. To date, it has been well addressed the correlation of single components or nutrients of the MD and the risk of oral pharyngeal cancer (OCP) [79]. However, only few studies have analyzed the impact of the MD pattern on the OCP cancer risk [80]. Specifically, Filomeno et al. reported that MD pattern reduced upper aerodigestive tract (UADT) cancer risk, whereas the impact of the individual dietary components of the MD was not sufficient to prevent UADT cancers, including oral cavity and oropharynx, larynx, and oesophagus cancers [80]. The protective role of MD in preventing OCP cancer is linked to the high intakes of foods enriched of biological compounds, which exert healthy effects. In fact, fruits and vegetables have reduced the risk of OCP cancer [81–83] because of their high content of carotenoids, vitamin C and E, as well as flavonoids [84]. In addition, EVO, enriched of antioxidant compounds including polyphenols, has a positive influence in reducing the risk of UADT cancers, including the OCP cancer [85]. Consistent with these data, dietary polyphenols have been found to prevent the periodontal tissue destruction maintaining the balance between oxidative stress and antioxidant activity in the oral cavity [86].

From the point of view of the development of carious pathology, with a multifactorial etiology, it has been demonstrated that MD is able to significantly reduce the appearance of carious lesions even at an early age [87]. Considering the relevant public health problem, to correlate the risk factors of tooth decay with a diet capable of limiting the acid action and fermentation of simple sugars by the oral microbiota, modulating it and making the systemic supply of protective factors constant represent a topic of great interest [88].

**Figure 2.**
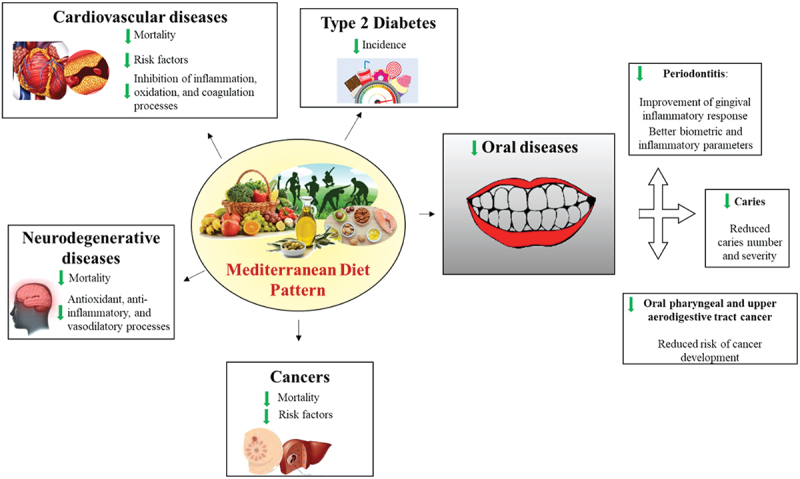
Impact of the MD pattern on the metabolic and chronic diseases, including oral diseases.

Despite the health benefits of the MD in the prevention of oral diseases, it is worth to note that regular oral hygiene practices, such as regular brushing and dental check-ups, are essential for maintaining oral health [89]. In this context, it has been observed that poor adherence to MD is associated with insufficient oral hygiene, characterized by higher bleeding on probing and plaque indexes, regardless to age, sex, physical activity, and diabetes mellitus as variables in multiple-regression analysis [89]. In addition, a positive association between the eating habits mirroring the MD food choices and healthy oral habits was found among kidney transplant recipients, which usually suffer from severe forms of periodontitis [90]. Specifically, patients with a higher MD adherence score had a higher number of teeth as well as an increased dental plaque removal [90]. These findings suggest that maintaining a proper dietary regimen, such as the MD, may contribute to improve oral health outcomes. Collectively, the health benefits of the MD on metabolic and chronic diseases, including oral diseases, are summarized in Figure 2.

## Etiology of oral diseases

Periodontal diseases are oral inflammatory diseases affecting the tissues supporting and surrounding the teeth and include gingivitis and periodontitis [91]. This same pathological condition, but in a clinically more serious and accelerated form, also occurs at the dental implant level, known as mucositis and peri-implantitis, similar to gingivitis and periodontitis, which significantly compromise the outcome of the rehabilitation therapy [92–94]. Although gingivitis is associated with bleeding, swollen gums, and pain in its progression, periodontitis is related to the loss of periodontal attachment and supporting bone, often asymptomatic [95]. However, if untreated, periodontitis can lead to tooth loss, with a consequent impairment in mastication function, esthetics, self-confidence, and quality of life [96,97]. The same important limitations can be caused by the progression and development of the carious pathology, which demonstrates a different multifactorial etiology compared to periodontitis and compresses the hard tissues of the tooth, often keeping the supporting bone and periodontal ligament intact [96].

The prevalence of periodontal diseases is estimated to range from 20 to 50% worldwide, emerging as the 11th most prevalent condition in the world as reported by the Global Burden of Disease Study of 2016 [29]. Heterogeneity among studies analyzed by Borg-Bartolo et al. among the different regions of the world was 99.7% (prevalence of edentulism) and 99.9% (prevalence of dental caries) [98]. The global estimated random-effects pooled prevalence of edentulism was 22%, which allows defining this pathology as a public health problem [98]. Based on this information, to understand what role the MD can have, it is necessary to understand which mechanisms underlie the etiopathogenesis of periodontal diseases and caries, also considering the different oral pathogens involved. Periodontal diseases have been shown to be related to low-grade systemic inflammation, potentially driven by several inflammatory mediators [91,99,100]. The possibility of studying the levels of peripheral tissue inflammation, before the signs of periodontal pathology are manifest (marginal bone loss, increased probing depth), represents to date the most important possibility for avoiding irreversible damage to the supporting tissues. In this context, early diagnosis performed through atraumatic analysis of mediators of peripheral tissue inflammation represents the main option to achieve an early diagnosis, and from this perspective, further studies are necessary to achieve an even more defined correlation relationship between the different markers of inflammation and clinical indicators of pathology [101]. Recent studies showed that patients affected by periodontal diseases are characterized by higher circulating levels of C-reactive protein, fibrinogen, neutrophils, and indirect systemic inflammatory markers, such as tumor necrosis factor and interleukin (IL) 1, IL-6, and IL-8 [102,103]. It is interesting to note that, in oral tissues, nutrition exerts a combined action, both at local and systemic level [104]. Excluding the process of formation of dental tissues, which can undergo significant alterations for various reasons and lead to conditions that are cariogenic, the caries process derives from the absorption of fermentable carbohydrates, including sucrose, glucose, fructose, lactose, maltose, and starch. These fermentable carbohydrates may have both local and systemic effects on dental caries, and these effects differ considerably depending on the conditions of the subjects, in particular depending on the age and the dental surfaces exposed (root/crown) [104]. Considering the complex action that macro and micronutrients carry out at a systemic level, the maintenance of periodontal health depends on the optimal nutrition with respect to both. Clearly, different foods, by local and systemic action, exert more complex interactions for the development of the carious process [105]. As gingival tissues are sentinel of overall systemic health, the absence of both dental caries and gum bleeding in the absence of oral hygiene could be considered a potentially sensitive indicator for an overall healthy diet [104].

## Oral microbiome in the development of oral diseases

Research has focused more on studying periodontitis and systemic diseases, exploring the role of oral inflammation and microbiota in conditions like atherosclerosis, diabetes mellitus, pneumonia, chronic obstructive pulmonary disease, rheumatoid arthritis, and Alzheimer’s disease [106–108]. While both dental caries and periodontitis are biofilm-mediated diseases, their pathogenesis differs. Dental caries involves multiple factors leading to localized demineralization of teeth, and the potential systemic consequences of untreated dental caries and the associated oral microbial-inflammatory process require further investigation through human and animal studies. The spread of the oral microbiome into the systemic circulation from dental caries is plausible, and parallel mechanisms could have observed in periodontal diseases. In dental caries, the root canal space or marginal periodontium are the most likely pathways for direct systemic extension of oral microbiota [9]. The oral microbial ecosystem is constantly exposed to exogenous substances, which concur to maintain the fine-tuned equilibrium of the oral health, or presents opportunities for oral microbial dysbiosis leading to oral and systemic diseases [109].

Starting from this point of view, dental plaque is defined as a biofilm composed of complex polymicrobial communities. This biofilm, responsible for various alterations at the dental level, receives most of its nourishment from the diet, which provides nutritional resources for the oral microbiota and serves also as a selective pressure by enriching for organisms best adapted to utilize specific host-derived dietary resources [110]. Major historical dietary shifts throughout evolution are accompanied by significant changes in the oral microbiota [111], which led to a selection of acid-producing and acid-tolerant organisms and periodontal pathogens [112]. Often, these bacteria represent pathobionts, capable of playing different and synergistic roles in the development of oral or systemic diseases depending on the general state of health of the host. It is also interesting to note how the conditions that modify the environment and the nourishment granted to the microbiome are different and vary throughout life, and in any case significantly during ageing [109]. It is possible to define carious or periodontal oral pathologies as a microbiome dysbiosis of the site under consideration, which can be caused by site-specific factors, systemic factors, or a combination of both [113]. Dental caries is particularly related to a high dietary intake of carbohydrates, leading to an increased production of acid by microbes (which hinder the buffering capabilities of saliva), a decrease in salivary pH, a greater production of biofilm exopolysaccharide matrix (that entraps and concentrates acids on enamel surfaces), and induction of positive-feedback loops that encourage outgrowth of aciduric and acidogenic species, including *Streptococcus mutans* and *Lactobacillus species* [113,114]. Although the evaluation of the microbiome involved in the etiopathogenesis of carious pathology is complex, that involved in periodontal pathology is even more complex, also considering the mutual interactions between the different bacteria it contains [115]. Taking into consideration for example the colonization by *Corynebacterium*, long filaments are developed on which distinct microenvironments are created, and streptococci at the periphery of oral biofilms produces lactate, which serves as a preferred substrate for catabolism by *Veillonella, Corynebacterium*, and *Eubacterium* species [113].

Dental caries is mainly caused by oral biofilm acid, and composite dental restorations are the most common treatment [116]. The main cause of failure is secondary caries adjacent to the restoration, and long-term survival of dental materials is improved by the presence of antibacterial agents, which selectively inhibit bacterial growth or survival [116,117]. Chemical, natural, and biomaterials have been studied for their antimicrobial activities and antibacterial bonding agents have been improved, in order to obtain an important antibacterial potential at the weakest interface of the restorative complex [118]. Through these new findings, it is possible to study characteristics, epidemiology, risk factors, clinical manifestations, diagnosis, and innovative treatments for several types of oral infectious diseases [119].

To date, we can define that the sub-gingival biofilm is also absolutely dependent on the biofilm found at the supra-gingival level, from which it is modified depending on the type of bacteria present and their metabolism depending on the external stimuli to which they are subjected [120]. Recently, it has been found that the *Bifidobacterium animalis subsp. lactis* (B. lactis) HN019 decreased the periodontal biofilm virulence, the gingival inflammation as well as the number of pathogenic bacteria characterizing the periodontal diseases [121–123]. In a randomized controlled trial, (*B. lactis*) HN019 reduced the bleeding in patients presenting with peri-implant mucositis, decreasing the levels of pro-inflammatory cytokines in the peri-implant crevicular fluid [124]. The bacteria that coexist at the periodontal level, including periodontopathogens, have been cataloged into different ‘complexes’, depending on their danger and effectiveness in altering the state of periodontal health [125]. To make this evaluation very complex, it is necessary to consider how, for example, *Streptococcus gordonii* has been shown to increase the virulence of *Aggregatibacter Actinomycetem comitans* by producing lactate via streptococcal carbon metabolism [126]. The potential interrelationships among microbes make it difficult to evaluate the behavior of a single pathogen, and definitively focus attention on the biofilm and the microbiome, a condition that significantly alters the response of some pathogenic species, which can even enhance the outgrowth of other pathogens through symbiotic relationships. This interplay demonstrates the role of microbial interactions in promoting diseases via multifaceted physical and metabolic interactions that underlie the polymicrobial synergy and dysbiosis model [127].

Due to the shortcomings of several antimicrobial medications frequently prescribed in dentistry, the lack of resources in developing countries, the prevalence of oral inflammatory conditions in diverse oral pathologies, and the ever-increasing growth of bacterial antibiotic resistance, there is a need for reliable, efficient, and affordable alternative solutions for the prevention and treatment of periodontal diseases [128]. Several accessible chemical agents can alter the oral microbiota, although these substances also have unfavorable symptoms, mainly caused by the imbalance of the oral/intestinal microbiota, such as vomiting, diarrhea, and tooth discoloration [129]. Precisely in light of this information, the natural phytochemicals generated from plants that have historically been used as medicines are categorized as prospective alternatives due to the ongoing quest for substitute products, ensuring greater compatibility of treatments [130]. A summary of the main virulence mechanisms of microorganisms most frequently involved in the development of oral diseases is reported in Table 1.

**Table 1.** Summary of the main virulence factors of the principle periodontal pathogens.

Microorganisms	Description	Associated Oral Condition	Ref.	
*Streptococcus mutans*	Primary bacterium linked to dental caries. Flourishes in the acidic environment, producing acids damaging tooth enamel	Dental caries		[131]
*Lactobacillus*	Contributes to dental caries by producing acids that erode dental enamel	Dental caries		[132]
*Bifidobacterium*	It can contribute to acid production and tooth enamel damage in dental caries	Dental caries		[133]
*Porphyromonas gingivalis*	Bacterium associated with gingivitis. Produces enzymes damaging gum tissues, leading to inflammation	Gingivitis		[134]
*Treponema denticola*	Associated with the progression of gingivitis, producing enzymes breaking down gum tissues and contributing to plaque formation/ Associated with severe periodontitis, produces enzymes contributing to gum tissue destruction and inflammation	Gingivitis/ periodontitis		[135,136]
*Tannerella forsythia*	Causes gum inflammation, generating toxins and enzymes that harm tissues, provoking an inflammatory response/ Implicated in the progression of periodontal disease by producing enzymes and toxins damaging gum tissues	Gingivitis/ periodontitis		[135,136]
*Aggregatibacter* *actinomycetemcomitans*	Linked to gingivitis and periodontitis, produces toxins and proteins damaging gum tissues/ Implicated in aggressive periodontitis, producing toxins causing tissue damage and bone resorption	Gingivitis/ periodontitis		[136,137]
*Fusobacterium nucleatum*	Commonly found in bacterial plaque, exacerbating gum inflammation and contributing to the formation of harmful biofilms/ Commonly found in periodontal pockets, co-aggregating with other pathogenic bacteria, contributing to the progression of the disease	Gingivitis/ periodontitis		[136,138]
*Prevotella intermedia*	Associated with gum inflammation, producing toxins that damage gum tissues and trigger an inflammatory response/ Associated with periodontal disease progression, produces enzymes and toxins contributing to tissue damage	Gingivitis/ periodontitis		[136,138]
*Porphyromonas gingivalis*	Bacterium associated with periodontitis. Produces enzymes and toxins leading to breakdown of gum tissues and bone.	Periodontitis		[136]
*Veillonella parvula*	Component of a specific bacterial complex observed in the microbial communities of subgingival plaque	Periodontitis		[139]
*Corynebacterium*	Component of a synergistic and dysbiotic microbial community triggering periodontal disease	Periodontitis		[113]
*Eubacterium*	Component of a synergistic and dysbiotic microbial community triggering periodontal disease	Periodontitis		[113]

## Potential role of MD in modulating oral microbiome in oral diseases

Many investigations focused on the discovery of the specific composition and microbial diversity, while only a few studies have investigated the impact that host genetics and environmental factors, including lifestyle, can have on the biological function of the oral microbiota. Based on the data reported in literature, diet could influence the composition of the oral microbiota and the development and progression of oral diseases [140].

So far, much of the research has focused on the impact of the MD in the gut microbiome, demonstrating that a high adherence to the MD leads to the microbiota eubiosis reestablishment, increased microbiota diversity as well as an improved gut-barrier function and permeability, decreasing the risk of chronic diseases such as cardiovascular diseases [141]. Recently, it has been reported that the MD positively affects resting metabolic rate and salivary microbiota enhancing the percentage of *Subflava* and *Prevotella* species with respect to Vegan Diet in humans [142]. Notably, the main difference between the two diet regimens is represented by lower protein intake in the Vegan Diet and by higher content of fibers in fruits and vegetables in MD, which explain the abundance of *Subflava* and *Prevotella* [142]. Particularly, some nutrients, such as fiber, positively influence the oral eubiosis suggesting the beneficial effects of the MD [143]. The oral cavity serves as a reservoir of *Staphylococcus aureus* for infection of the lower respiratory tract and cross-infection, suggesting that *Staphylococcus aureus* continues to be a frequent isolate in the oral cavity and perioral region [144]. Thus, the role of *Staphylococcus aureus* in the pathogenesis of certain oral diseases should also be considered as part of a complete differential diagnosis [145].

Moreover, it has been found that the consumption of foods and beverage characterized by a high content of polyphenols has antimicrobial activities on oral pathogenic bacteria such as the *Staphylococcus aureus* and *Porphyromonas gingivalis* [146,147]. However, some studies have primarily examined the effects of specific micro or macro-nutrients from the MD on the microbiome, rather than considering the overall dietary pattern. The association between micro and macronutrients and the main microorganisms is summarized in Table 2.

**Table 2.** Associations between nutrients and oral microbiome.

Dietary sources	Nutrients	Associated microrganisms	Ref.
**Micronutrients**			
Meats, fish, and whole grains	Vitamin B1	*Neisseriaceae* *Gemellaceae* *Fusobacterium nucleatum*	[148–151]
Meats, green vegetables, dairy products	Vitamin B2		[149,150]
Eggs, fish, meat, mushrooms, nuts	Vitamin B3		[149,150]
Beef, chicken, organ meats, broccoli, avocados	Vitamin B5		[150,152,153]
Meat, vegetables, nuts, banana	Vitamin B6		[150,154]
Leafy vegetables, peanuts, raw egg, liver,	Vitamin B7		[150,155]
Leafy vegetables, cereals, legumes, nuts and seeds	Vitamin B9		[150,151]
Fish, meat, poultry, eggs, and dairy products	Vitamin B12		[150,156]
Green vegetables (kale, spinach, cabbage, lettuces), egg yolk	Vitamin K	Not identified	[157]
Fish eggs, liver, mushrooms, milk	Vitamin D	*Fusobacterium nucleatum, Porphyromonas gingivalis*, *Pseudomonas aeruginosa*, Candida *spp.*	[150,158–162]
Citrus fruits, fruits (oranges, kiwi, lemon, grapefruit), kiwis and strawberries; Cruciferous vegetables (broccoli, brussels sprout, cabbage, cauliflower); tomatoes, bell peppers and white potatoes	Vitamin C	*Neisseriaceae* *Lepto-trichiaceae* *Lachnospiraceae* *Fusobacterium nucleatum*	[74,148,163–167]
Dark leafy greens vegetables, egg yolk, vegetable oils, nuts, sunflower seeds, pumpkin	Vitamin E	*Neisseriaceae* *Fusobacterium nucleatum Gemellaceae*	[74,148,163–168]
	Vitamin A	Not identified	[74,164]
Milk, dairy products, fish, eggs, leafy vegetables, nuts, seeds	Calcium		[169,170]
Green leafy vegetables, legumes, nuts, seeds, whole grains	Magnesium	Not identified	[169]
Grapefruits, nuts cocoa, tea, dried fruits, oatmeal	Fluoride		[171,172]
Meat, dry beans, spinach, shellfish	Iron		[173]
Fish meat, seafood, spinach, grains	Zinc		[174,175]
**Macronutrients**			
Fish, meat, milk, dairy products, eggs and pulses	Proteins	Not identified	[176–179]
Cereal and derivates (pasta, rice, bread) potatoes, legumes, vegetables, fruits	Carbohydrates	*Lacto-bacillaceae* *Fusobacterium nucleatum*	[104,148,180–184]
Oily fish (salmon, sardines, mackerel, herring, cod liver oil); Vegetables (olive oil, oilseeds, nuts grape seed oil, soya oil, sunflower oil); Butter, cheese, yogurt	Fats	*Fusobacteria nucleatum* *Neisseriaceae*	[74,148,161,164,185]

Despite these findings that support the potential positive effects of the MD in modulating the oral microbiome, our understanding still remains limited. The wide variability in genetic and environmental factors including dietary habits and lifestyle represents a limitation for study design and finding interpretation making difficult to enhance our knowledge in this field. Specifically, further research will be needed to shed light on the mechanisms by which nutrition protects oral microbiome eubiosis maintaining oral health.

## Conclusions

A healthy lifestyle, based on the Mediterranean model, impacts on the general health status of human beings, including oral health. Overall, MD is rich of anti-inflammatory and antioxidant compounds, which may induce beneficial effects in patients suffering of oral diseases, mainly represented by dental caries and periodontal diseases and also positively affects oral microbiome. Conversely, an unhealthy diet affects the composition of the oral microbiota, leading to dysbiosis. To date, an interesting and unexplored area regards the potential link among MD, oral diseases, and oral microbiome, which deserves to be further investigated.

The dentist plays a fundamental role in promoting and disseminating the correct dietary habits based on healthy food choices among the population that, together with lifestyle, may significantly improve their general and oral health status.
